# Low-Cost Portable System for Measurement and Representation of 3D Kinematic Parameters in Sport Monitoring: Discus Throwing as a Case Study

**DOI:** 10.3390/s22239408

**Published:** 2022-12-02

**Authors:** Juan Francisco Navarro-Iribarne, David Moreno-Salinas, José Sánchez-Moreno

**Affiliations:** Department of Computer Science and Automatic Control, National Distance Education University (UNED), C/Juan del Rosal 16, 28040 Madrid, Spain

**Keywords:** sports wearable, monitoring, kinematic parameters, embedded portable system

## Abstract

Monitoring of sports practice has become an almost essential tool in high-level professional training. The knowledge of the exact movements performed by an athlete provides a great advantage over conventional training, since the best performance can be theoretically known in advance and the trainer will expect the real athlete’s movements to approximate it. Following this trend, this article deals with the design and development of a low-cost wearable biofeedback system for the measurement and representation of kinematic parameters in 3D. To capture the athlete’s movements, an inertial measurement unit (IMU) is used, whose data are processed in an microcontroller-based architecture. The kinematic parameters of the athlete’s movement are sent via Bluetooth to a smart phone, where they are displayed graphically. Experimental examples show the effectiveness of the device developed and illustrate the key results derived.

## 1. Introduction

Human physical monitoring in personal and professional activities has been increasing over the last few years, due both to the growing interest in a healthy life style and the search for the improvement of professional athletes’ training. This interest, combined with recent advances in sensors, wireless communications, and embedded systems, has enabled the development of smart and intelligent devices to monitor human and sports activities in real-time [1,2].

The training of an athlete is based on two fundamental pillars, the physical one and the technical one, with both impacting the tactical side of the sports. Over the years, the increasing competitiveness and professionalization of sports have made it necessary to update procedures to improve the athletes’ results. One of the greatest allies for improving the technical training is technology. Since it began to be part of our lives, technology has been used in multiple areas, including athletes’ training. A concrete example and one of the first applications of modern technology in sports is the camcorder. When athletes want to improve their technique, it is very useful to know what they are doing wrong to correct it. The video camera allows recording the sequence of the movements performed and studying them afterwards to improve the technique [3]. This system has the drawback that it only offers visual information of the athlete in a two-dimensional plane. Some studies on this matter have used two home cameras to obtain a three-dimensional representation, but the results have not been very promising, since, although the movement of the athlete is captured, there is no precise quantification of the parameters involved. Moreover, the information is not analysed in real-time, and then, it cannot be used by the athlete or coach during the training.

Following this trend, biofeedback systems are among the most promising applications of technology in sports. These systems include sensors, processing devices, actuators, and other elements that process the information gathered and present it to the athlete in some way during the training. This feedback can be used to react adequately according to the information received; see for example [4], where sensors, actuators, and wireless technologies are studied and described for their use in biofeedback systems, or [5], where a real-time monitoring of sports is presented.

These kinds of systems may be used to provide feedback to the athletes or monitoring for coaches and trainers in a wide variety of sports and activities. For example, in [6], the real-time acquisition of kinematics parameters during the practice of Nordic walking was performed. A wireless sensor network is embedded into the sports equipment to monitor the athletic gesture, providing real-time biofeedback to identify and prevent common errors in the sport’s practice. In [7], a wearable body sensor network measures the wrist rotation in golf swing for improving the quality of training, or in [8], the heart rate is measured and monitored for the practice of archery. The work in [9] presents the design of an embedded system for volleyball assistant decision support based on association rules, and in [10], an inertial sensor carried on the wrist is used to monitor the training and game sessions of basketball players. Even some platforms for sports training and education have been developed, such as in [11], where an embedded system and computer technology are used to develop a virtual reality sports auxiliary training system to improve the training effect and physical education teaching.

In addition, technology can also be used to prevent sports injuries by detecting dangerous practices. For example, an injury prediction model is presented in [12] based on an embedded system and a machine learning algorithm. An artificial neural network (ANN) was trained using information from soccer players to detect those in danger of injury. The technology, apart from being an aid to athletes and coaches, can be also used to avoid tedious activities related to the sport’s practice; see for example [13], where a robot is designed to recover the tennis balls used during training, avoiding the intervention of human operators.

Notice that all the devices described above were developed to be used in sports, but they could also be applied to more general issues, such as human activity monitoring to improve life conditions. In this sense, there exists a wide literature about devices designed to measure, control, and monitor everyday gestures. See for example [14], where a hardware framework is designed to detect the falls of elderly people using inertial sensors and compressed sensing, or [15], where an autonomous embedded device, composed of a low-latency real-time location system (RTLS) and a low-cost IMU, is designed for guiding blind people using a specialized virtual sound source.

The video recording of the movements made by athletes in any sports discipline and their subsequent reproduction for analysis can allow certain aspects of their technique to be improved, but there is no quantification of the parameters involved in the dynamics of the movement. There are mainly two parameters, and they must be measured during a specific moment of the activity carried out by the athlete: trajectory made during the execution of an exercise and movement speed.

In the present work, the monitoring and biofeedback focus on the kinematic parameters of the athlete’s hand for the discus throwing sport [3]. There are several works that try to study and/or monitor the discus throwing activity in order to improve the technique and results. In this sense, in [16], the comparison of the kinematics of movements of the arm between standing and rotational discus throws is studied. A temporal analysis of the discus throwing technique is carried out in [17]. In particular, the relationship between the duration of the throw and the throwing distance is studied for the evaluation of the discus throwing technique. In [18], the kinematic characteristics of the jumping discus throwing technique are analysed; the analysis was carried out with two high-speed digital cameras to capture the movement, showing that this kinematic analysis may be used to improve the jumping discus throwing technique by athletes and coaches. Finally, in [19], individualized optimal release angles for elite discus throwers are studied. The regression relationship between release speed and angle and between distance and release angle were determined for each thrower; the optimal release angle was estimated for each athlete, showing that this angle is thrower-specific.

The article at hand describes the design and construction of a low-cost device to monitor and track the hand and wrist movements in discus throwing, providing biofeedback to the user by a smart phone application. There are mainly two parameters to monitor: trajectory and speed of the athlete’s hand movement. Then, the main contributions of the present paper are threefold: (i) a wearable embedded system composed of a low-cost IMU and a microcontroller is designed and constructed to measure and represent the kinematic parameters in 3D of the athlete’s movements; (ii) the algorithms that allow the correct filtering and adaptation of the variables collected by the IMU are defined and described; and (iii) the information gathered by the device is sent to a portable computer system (a smart phone) for graphical representation and analysis via Bluetooth wireless communication.

Notice that the purpose of this work is to describe a low-cost solution to record the technical parameters of a specific body landmark, not to provide a bio-mechanical study of elite or amateur athletes by analysing the relationship between performance and kinematics parameters. Another important feature of the solution presented is the recording and plotting of 3D trajectories in real-time of some kinematic parameters of a body part (in our examples, the athlete’s wrist), which is not present in typical high-speed video recording and data processing systems used in the sports literature [3,19,20,21,22,23,24].

The rest of the paper is organized as follows. In Section 2, the system components and main tools used in the device’s construction are presented, and the general description of the prototype designed to be worn on an athlete’s limb is given in Section 3. In Section 4, both the programming of the wearable device and the smart phone app are described. The system validation and experimental tests are shown in Section 5. Finally, the conclusions and future works are described in Section 6.

## 2. System Components

### 2.1. Inertial Measurement Unit

Inertial measurement units (IMUs) are composed by a set of sensors used to acquire several physical variables of a moving body. These sensors are based on microelectromechanical systems [25]. They are divided into: accelerometers, which measure linear acceleration in each of the axes; gyroscopes, for angular velocity around the axes; and magnetometers, to measure the strength of the Earth’s magnetic lines that cross each of the axes.

In addition to these physical variables, it is necessary to process the information gathered to determine the orientation and speed of the device to be monitored, and it is usual to use microcontrollers to manage the information that the IMU provides.

### 2.2. Fusion Algorithm

The main objective of data fusion algorithms is to improve the quality of the information obtained from processes with multiple sensors, known as synergetic, since they act together for a common goal [26]. Using more than one sensor not only increases the spatial and temporal coverage of the variables studied, but it also increases the suppression of noise and the accuracy of the measurement system. There are several filtering methods to fusesensor data, each with different degrees of complexity, and the one discussed in this work is the Madgwich filtering method [27]. This algorithm uses a representation of the orientation by means of quaternions, which are free of the singularity problems present in representations based on direction cosine matrices. The algorithm uses the gradient descent method to calculate the direction of the gyroscope measurement error.

### 2.3. Euler Angles vs. Quaternions

It is possible to represent the rotation of an object in space in different ways. Euler angles are the easiest visualization method. This representation is composed of three components (yaw, pitch, and roll), and it has many limitations, the main one, and perhaps the most important, being the gimbal lock. This phenomenon occurs when 2 or 3 axes of rotation are parallel or very close to being parallel, decreasing the degrees of freedom of the system. At that moment, one degree of freedom is lost and the information regarding the involved axis disappears. When the gimbal lock appears, it is impossible to reorient the axes without an external reference.

To avoid this problem, the use of quaternions is recommended. Quaternions can be used as a mathematical tool to work with rotations and orientations with great computational versatility [28].

A quaternion *Q* is made up of four components $\left(q_{0},q_{1},q_{2},q_{3}\right)$, which represent the coordinates of the quaternion in a base e,i,j,k. It is common to name the component $q_{0}$ as the scalar part of the quaternion and the rest of the components, $\left(q_{1},q_{2},q_{3}\right)$, as the vector part.

In this way, a quaternion can be represented as:

(1) $$Q=\left[q_{0},q_{1},q_{2},q_{3}\right]=\left[s,v\right]$$

where *s* represents the scalar part and v the vector part. For the use of quaternions as a methodology for representing orientations, the rotation of an angle θ about the vector k is associated with the quaternion defined by:

(2) Q=Rot(k,θ)=(cosθ/2,ksinθ/2)

Quaternion composition is as simple as multiplying quaternions. In such a way, the result of rotating according to the quaternion $Q_{1}$, to subsequently rotate according to $Q_{2}$, is the same as that of rotating according to a quaternion $Q_{3}$ obtained by the expression:

(3) $$Q_{3}=Q_{2}\cdot Q_{1}$$

It is important to keep the order of multiplication, since the product of quaternions is not commutative. The use of quaternions for the composition of rotations is a very practical computational method, since it is enough to multiply quaternions among themselves, which corresponds to a very simple expression of products and sums.

### 2.4. Bluetooth Low Energy

Bluetooth Low Energy (BLE) was introduced as part of the Bluetooth 4.0 specification. Although there is some overlap with classic Bluetooth, BLE comes from a project initially developed by Nokia and known as “Wibree” before it was adopted by the Bluetooth Special Interest Group (SIG). It is also known as Bluetooth Smart. There are several wireless protocols for use in the Internet of Things (IoT), but BLE is the easiest way to implement communication between small devices and an application on any operating system (iOS, Android, Windows). Particularly in the case of iOS devices, it is the only method that allows the interaction of peripherals with applications without the need for Made For Iphone (MFI) certifications and other legal requirements required for iOS.

## 3. System Description

**Figure 1:**
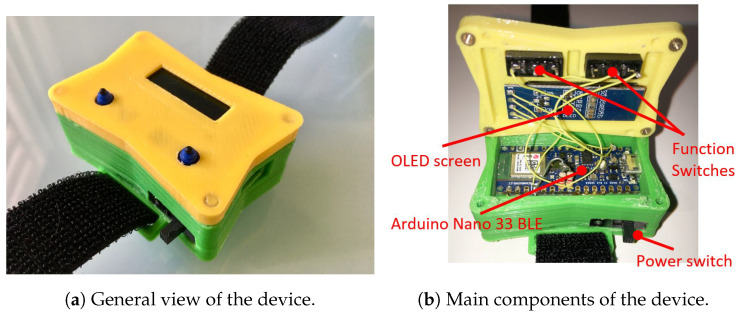
Exterior (**a**) and interior (**b**) view of the device.

Instead of analysing the movement of the athlete’s entire body, the analysis will focus on the movement performed by a limb, preferably a hand or an arm. This analysis was carried out using the device presented in this article, which allows the capture of the necessary physical variables during the athlete’s movement to subsequently visualize these variables graphically. The acquisition was made using low-cost electronics, and its central core was an Arduino board. This board incorporates, among other devices, an inertial measurement unit (IMU), which is responsible for capturing the physical variables needed for the subsequent processing of the movement, a Bluetooth Low Energy communication module to transfer the measurements to a smart phone, and a battery, which provides the device with the necessary power for its operation in a portable way. All these components were placed in a casing manufactured for this purpose, which is preferably placed on the athlete’s wrist; see Figure 1.

**Figure 2:**
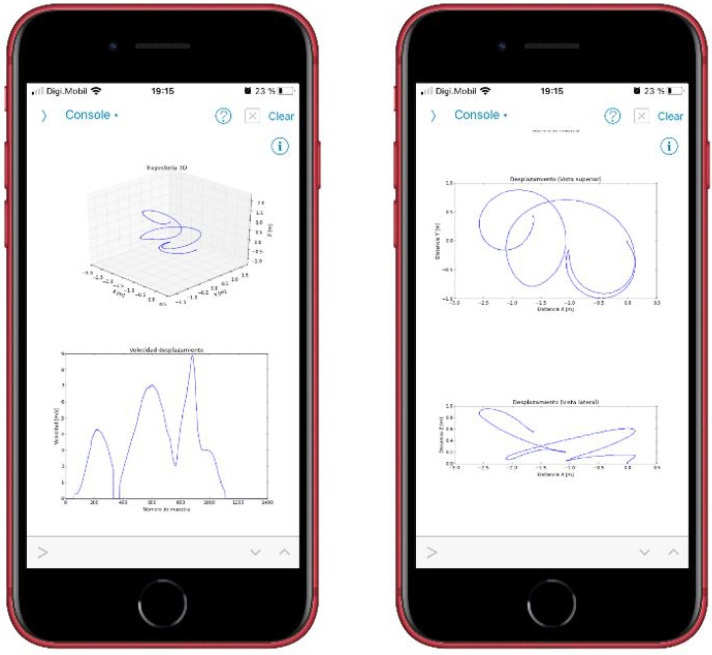
Graphical representations on a smart phone.

Once the phase of acquiring, processing, and sending the information to the smart phone is over, the athlete or any person in charge of the training will be able to see the trajectory and speed of the movement performed. See Figure 2 for an example of the movement representation of a discus throw on the smart phone.

**Figure 3:**
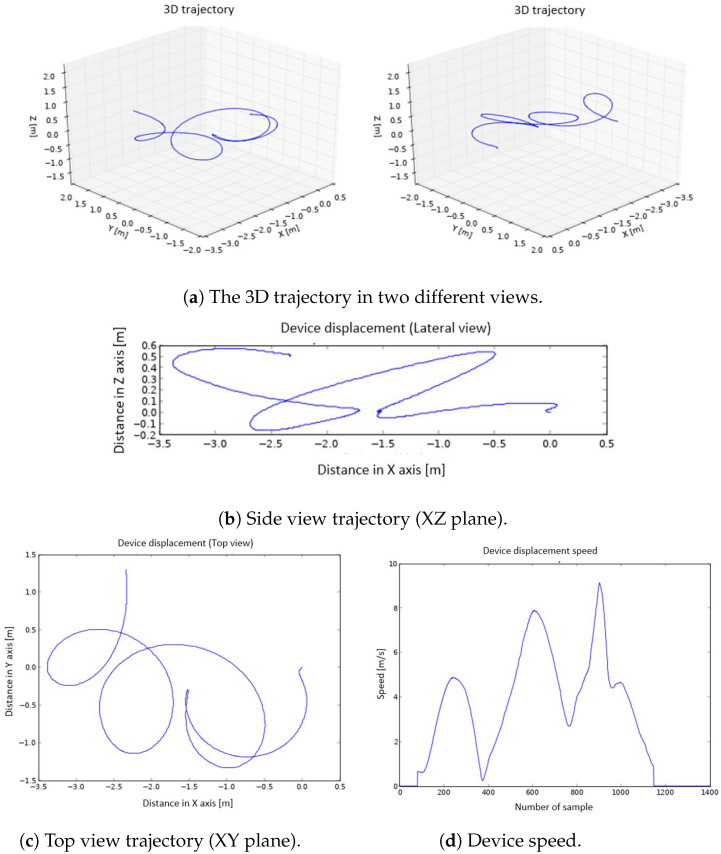
The 3D graphs (**a**), side view (**b**), top view (**c**), and device speed (**d**) representations.

The graphs generated on the smart phone are:

- The 3D representation of the trajectory (Figure 3a).
- The 2D representation of the trajectory seen from a side view (XZ plane) and from above (XY plane) (Figure 3b,c).
- The representation of the speed (Figure 3d).

The total cost of the device is estimated to be, approximately, EUR 60: Arduino Nano BLE (EUR 22), rechargeable 3.7 V Li-Pol battery of 500 mAh (EUR 5), battery charge controller (EUR 6), vibration motor (EUR 2), 0.91″ OLED screen (EUR 5), two micro-switch buttons (EUR 20), and some electronics components (EUR 10). The casing was manufactured by a 3D printer, and due to its size, the cost was very low.

## 4. Methodology

The development of the software was carried out in two different environments. The first one was for the programming of the microcontroller that will receive the information from the IMU, process it, and send it to the smart phone, and the second one was for the programming of the smart phone app to represent graphically the information received from the microcontroller.

### 4.1. Programming the Microcontroller

The program for the microcontroller located on the Arduino Nano 33 BLE board was written in C++ under the “Visual Studio Code” programming environment using the “PlatformIO” extension. This platform allows the programming of a multitude of microcontrollers, among which is the nRF52840 of the Arduino Nano 33 BLE board.

#### 4.1.1. Program Structure

The development of the program that receives, processes, and sends the information from the IMU to the smart phone is divided into two blocks. One is dedicated to the reception and processing of the information received from the IMU, and the other deals with the sending of the information already processed to the smart phone via Bluetooth. There is a common item to both blocks, the OLED screen. The screen uses the SSD1306 chip, and it is managed with the Arduino library “SSD1306Ascii.h”. This screen shows information for each of the phases of the program.

#### 4.1.2. Reception and Processing of Information from the IMU

The IMU used in this work is the one installed on the Arduino Nano 33 BLE board. Specifically, it is the LSM9DS1 chip from the manufacturer ST. It has 9 degrees of freedom, 16 bits of resolution, and is connected to the microcontroller through the $I^{2}C$ protocol. The use of the Arduino“LSM9DS1.h” library allows controlling all the resources of the IMU:

(a) Configuration of sensors. Some of the IMU parameters must be established before its use to allow the correct operation of the sensors.

(b) Calibration parameters. The offset and slope values are indicated so that each of the 3 sensors is correctly calibrated [29]. The calibration process is described in Appendix A.

(c) Full scale. It is necessary to establish what will be the maximum magnitude that each of the sensors can read, since this value entails an increase or decrease of the measurement resolution. Table 1 shows the full scale obtained empirically for each sensor.

(d) Output data rate (ODR). This parameter establishes the refresh rate of each sensor. Given the acceleration to which the IMU is subjected and the rapid changes in direction it can undergo, it is necessary for the sensors to have a refresh rate as high as possible. The set values are shown in Table 2.

#### 4.1.3. Control Structure

**Figure 4:**
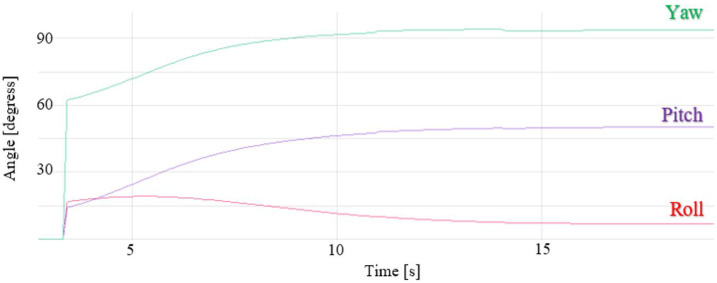
Euler angles’ representation during the stabilization phase of the measurements.

The information capture and its processing are managed by a “switch … case” control structure, with the program divided into 4 phases. The objective of dividing the program into several phases is, in addition to its order and clarity, to allow as much samplings as possible during the IMU data capture phase, achieving a higher-quality result. The sampling time is 0.002 s. It is important that the program only spends time in reading information from the IMU during the capture phase and it does not waste time on unnecessary tasks:

(A) **Phase I**: Standby mode. When the device is turned on by the power switch, the device goes into standby mode. To exit this phase and move on to the next one, the button on the left of the casing must be pressed; see Figure 1a. During this phase, the message “MOLADIS” is displayed on the device screen.

(B) **Phase II**: Conditioning. After pressing the left button, a countdown appears on the screen. It is necessary to wait some time for the calculations made by the fusion algorithm to stabilize the measurements; see Figure 4. The algorithm uses the gradient descent method to compute the direction error of the gyroscope measurements. Then, the initial values provided by the fusion algorithm are not precise, diverging from the real ones, so it is necessary to wait some time until the estimation error is reduced and stable and precise values of the angles are obtained. A value of 20 s was set to ensure that the computed values are stable.

**Figure 5:**
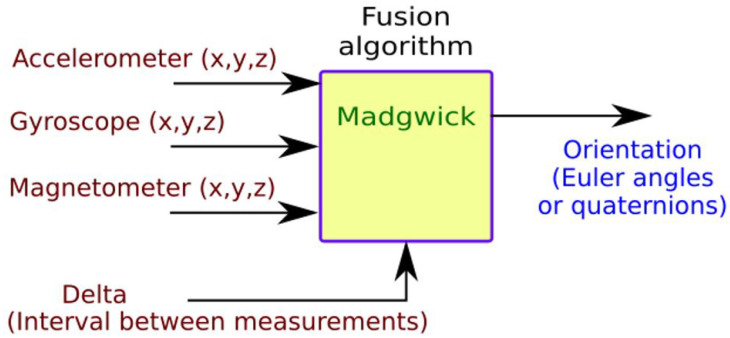
Fusion algorithm.

**Figure 6:**
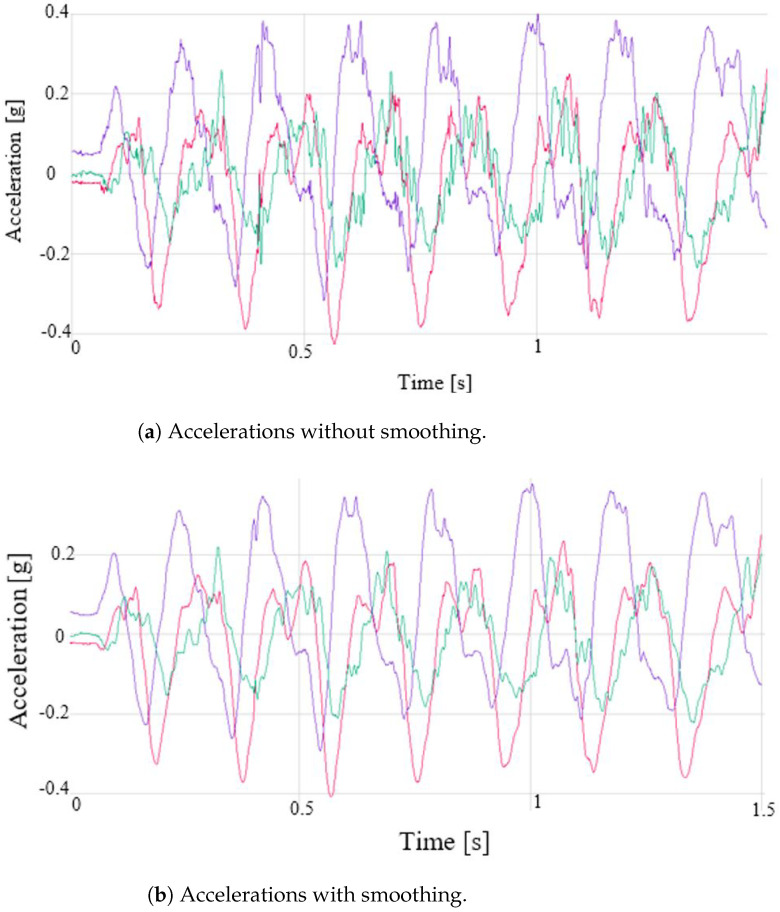
Raw (**a**) and smoothed (**b**) acceleration signals in X-axis (red), Y-axis (green) and Z-axis (purple).

**Figure 7:**
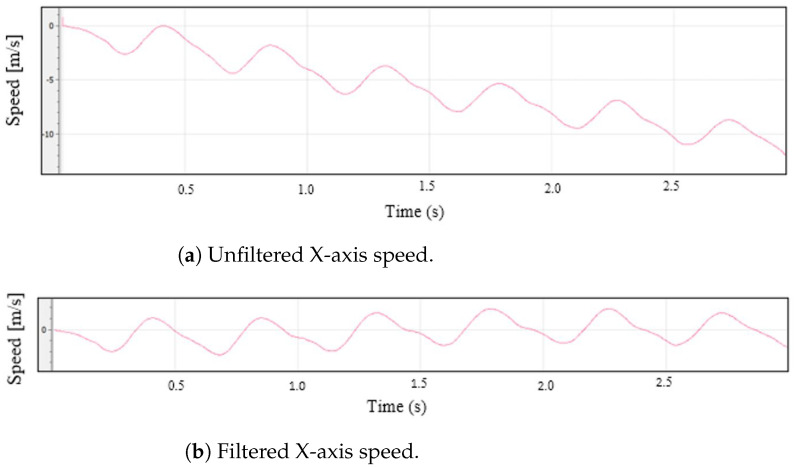
Unfiltered (**a**) and filtered (**b**) X-axis speeds.

**Figure 8:**
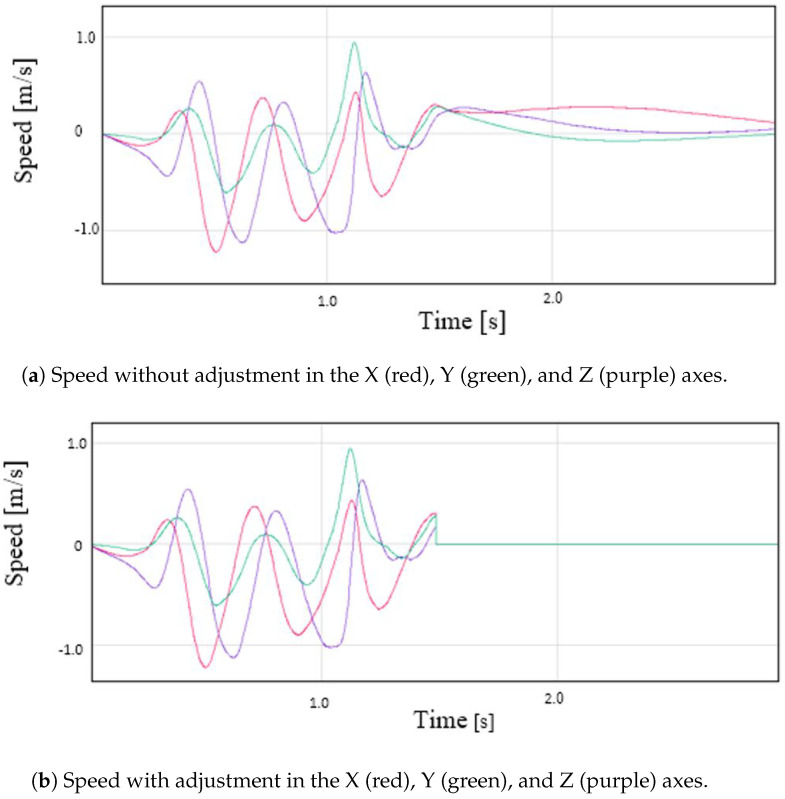
Speed without (**a**) and with (**b**) adjustment in the X (red), Y (green), and Z (purple) axes.

To graphically represent the stabilization of the calculations, the yaw, pitch, and roll parameters were used. Although they are not used in the subsequent calculations, since the computations are performed in quaternions, they are valid to represent the time required by the fusion algorithm for their stabilization. Once the timing is over and just before the program moves on to the next phase, the vibrator motor is briefly activated for 500 ms to indicate that the capture phase is about to start.

(C) **Phase III**: Capture. The capture process is based on the collection of data from the IMU. In this case, a capture of 3000 instantaneous values is made, which are stored in several arrays: 3 arrays for accelerations (accelerometer readings in X, Y, Z), 3 arrays for the gyroscopes (gyroscope readings with respect to X, Y, Z), 3 arrays for the magnetometers (magnetometer readings in X, Y, Z).

(D) **Phase IV**: Processing. In this phase, the computation of the speed and position of the device is made given the IMU measurements obtained in the capture phase (accelerometer, gyroscope, magnetometer measurements). A processing operation is performed for each of the measurements. This phase is also divided into several stages:

1. Data fusion. All the measurements collected at a given moment are combined using the Madgwick fusion algorithm to improve the quality of the output information. Figure 5 shows a schematic of its operation.
  It should be noted that for the correct operation of this fusion algorithm, the north, east, down (NED) convention must be followed: X-axis aligned with north, Y-axis aligned with east, and Z-axis pointing down.
2. Quaternions. To prevent the gimbal lock problem, quaternions instead of Euler angles are used. The use of quaternions reduces the computation time, since it avoids trigonometry calculations.
3. Suppression of gravity. An accelerometer is subject to dynamic accelerations (due to the athlete’s movement) and static ones (gravity). In order to obtain the correct measurements, it is necessary to eliminate the static acceleration. Then, the expected direction of gravity is calculated and then subtracted from the accelerometer readings. The result is the dynamic acceleration.
4. Rotation matrix. The IMU will always have, at least, one of its axes (IMU’s reference frame) rotated with respect to Earth’s reference frame. Thus, it is necessary to rotate it. The rotation matrix with quaternions is implemented as shown in (4) (Kong, 2002):
  (4) $$R\left(q\right)=\left(\begin{array}{ccc} q_{0}^{2}+q_{1}^{2}-q_{2}^{2}-q_{3}^{2} & 2q_{1}q_{2}-2q_{0}q_{3} & 2q_{1}q_{3}+2q_{0}q_{2}\\ 2q_{1}q_{2}+2q_{0}q_{3} & q_{0}^{2}-q_{1}^{2}+q_{2}^{2}-q_{3}^{2} & 2q_{2}q_{3}-2q_{0}q_{1}\\ 2q_{1}q_{3}-2q_{0}q_{2} & 2q_{2}q_{3}+2q_{0}q_{1} & q_{0}^{2}-q_{1}^{2}-q_{2}^{2}+q_{3}^{2} \end{array}\right)$$
  The accelerations with respect to the Earth’s reference frame are computed by multiplying the inverse of (4) by the accelerations to which the device is subjected. In this way, the gravitational acceleration is eliminated.
5. Acceleration filtering. The acceleration signal obtained by the IMU is a signal with noise and some glitches that may generate errors in subsequent calculations. To obtain a smoother signal, an exponentially weighted moving average (EWMA) noise reduction filter is applied. This filter has a low computational cost, and it is based on taking *N* samples, adding them, and finally, dividing the result by *N*. The average is “moving”, so it is recomputed every time that a new sample is obtained by taking the previous *N* samples. The value of N was fixed to 50, and the approximated cutoff frequency of the filter was 115 Hz. Figure 6 shows a raw and a smoothed signal.
6. Speed calculation. Using the filtered accelerations of the previous phase, the instantaneous speed at each sampling time is calculated by integrating the accelerations. The integral computation is carried out by dividing the area under the acceleration curve into rectangles, whose sides are instantaneous accelerations and the time interval between measurements, and adding the area of all of them. The result of this operation is the device speed [30].
7. DC filtering. The speed computation from the integration of the acceleration measurements entails a problem. Since the result is calculated as the cumulative sum of partial areas, if there is an error in any of these areas, caused, for example, by noise in the signal, the error will affect the result. If the noise causes errors in several areas, the result will differ quite a bit from what is expected. This fact is easily appreciated when the acceleration of a mobile is integrated to calculate its speed. If a movement of the stop–start–stop type is monitored, it is possible that the final computed speed is not exactly 0 (although the mobile has really stopped). If these errors are not corrected or minimized, in subsequent position calculations, the results would be quite incoherent. One way to reduce this problem is to eliminate the DC component from the velocity signal by applying a high-pass filter. The value of the cutoff frequency was determined empirically by the results obtained in the calculation of the device displacement. It is convenient that the cutoff frequency is not very high, since this causes the continuous component elimination, cancelling any possible representation of the displacement of the device. A low cutoff frequency causes the filter to have little effect on the output signal. The cutoff frequency was set to 50 Hz, and the sampling time was 0.002 s. The graphs in Figure 7 show the effectiveness of this filtering stage, where the speed in the X axis is shown, both unfiltered and filtered.
8. Zero speed. The acceleration read by the IMU, even if it is completely stopped, will never be 0.0, but it has a small value. This value, no matter how small, ends up impacting the calculated speed. In order for the computed speed to be 0.0 when the device has almost stopped, a small adjustment is applied. If the measured acceleration is within a window for a set of consecutive samples, the speed for the entire period will be 0.0. For the device, the window limits were +0.3 and −0.3 m/s${}^{2}$, and the number of samples considered was 60. Figure 8 shows the operation of this algorithm.
9. Speed calculation. One of the objectives of this work is to calculate the speed with which the athlete’s hand moves. In the previous step, the speed in each of the axes was computed, so the value of the speed for each time instant is:
  (5) $$Speed_{\left(t\right)}=\sqrt{V_{x(t)}^{2}+V_{y(t)}^{2}+V_{z(t)}^{2}}$$
10. Position calculation. Similar to what was done for the calculation of the velocities from the accelerations, the positions are computed by integrating the velocity. This process is carried out at each instant of time and for each axis. Unlike the calculation of the speed, the results obtained for the position should not be filtered, since it would directly impact the result. If, for example, a filter is performed to eliminate the continuous component, a possible displacement that was being carried out may be erased.

(E) **Phase V**: Waiting for the Bluetooth connection. The next phase after the movement computation is to send the information via Bluetooth. Before sending it, it is necessary to configure the communication between devices.

#### 4.1.4. BLE Configuration

**Figure 9:**
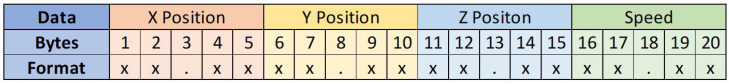
Data packaging. A 5-byte float with the format xx.xx is sent for each position coordinate and the speed.

The BLE configuration requires several steps:

1. Waiting for the connection from the central device. The program will be stopped at this point until the smart phone (central) establishes communication with the device (client).
2. Data packaging. One of the limitations with BLE is that, unlike conventional Bluetooth serial transmission, a large frame of characters cannot be sent. BLE only allows 20 bytes to be transmitted in each frame. To make the most of each frame and the transmission of all the information last as short as possible, the packaging shown in Figure 9 was used.
  The frame is constructed by concatenating the values of the positions in the 3 axes and the speed for each of the measurements. Once each frame is received by the smart phone, it is decoded following the previous packing scheme.
3. Sending data to the smart phone. The frames are sent to the smart phone as soon as they are generated. The total number of frames sent corresponds to the number of samples taken by the IMU, in this case 3000 samples.

### 4.2. Programming on the Smart Phone

The smart phone program, which receives the information sent by the device via BLE and represents graphically the movement and speed of the athlete’s hand, was written using the Python programming language. For iOS devices, there is a programming environment called Pythonista, which allows the editing and execution of any program written in Python. Specifically, this program was written and executed on an iPhone 6S. The program is divided into two blocks: data reception and graphic representation.

#### 4.2.1. Data Reception

The functions for receiving data via BLE are obtained from the Core Bluetooth Module (“cb” module) loaded at the beginning of the program. The smart phone will act as the central device in the BLE transmission. For this reason, it must scan the visible devices (peripherals) that are within its reach and pair with a specific one, the device. The phone will recognize it by its assigned name. Subsequently, the control panel must search for the service with the Universally Unique IDentifier (UUID) assigned to the device, and once found, it will search for the characteristic whose UUID was also established on the device. Every time that a data packet is received from the device, the 20-byte data frame is delimited and each of the 4 received variables are separated and stored in independent arrays.

#### 4.2.2. Graphic Representation

For the graphic representation, the module called “matplotlib” is used. This module allows the representation of values stored in arrays both in 2D and 3D.

**Figure 10:**
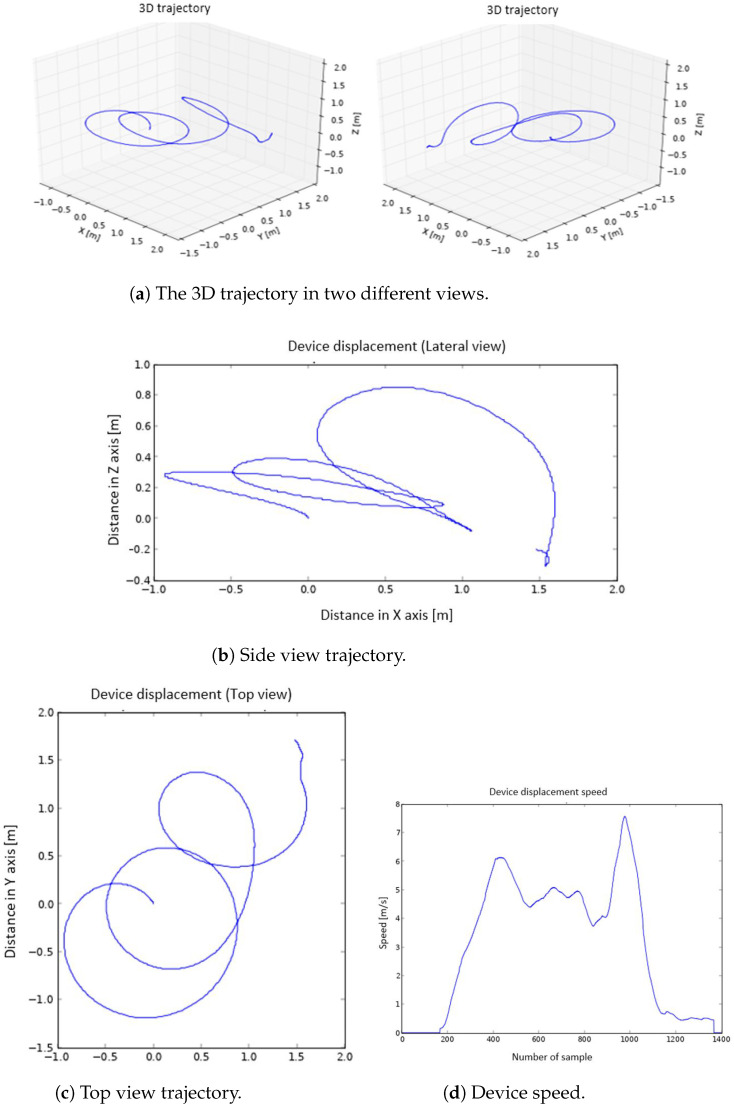
The 3D graphs (**a**), side view (**b**), top view (**c**), and device speed (**d**) representation.

To represent the trajectory followed by the athlete’s hand, a 3D graph is used. The program shows the same trajectory from different points of view, in order to be able to select and save the one that offers the best perspective. The beginning of the movement is at point (0,0,0). A set of representations of the same movement is shown in Figure 10. The trajectory is also represented in 2D, to make it easier to visualize. Specifically, it is displayed in a top view (XY plane) (Figure 10c) and in a side view (XZ plane) (Figure 10b). To represent the speed, a 2D graph is used (speed vs. sample number) (Figure 10d).

## 5. Device Operation

As mentioned above, the objective of this work is the graphical representation on a computing system of the trajectory and speed of the athlete’s hand during the practice of a sport or physical activity. The use of this device for sports monitoring is valid for all those sports in which a specific and repetitive movement is carried out. For example, it could be used to monitor a ball hit during the serve in volleyball or in various throwing disciplines in athletics. Specifically, this device was made for the discus throw, and a 1 kg plastic discus was used by a discus thrower for the different tests.

### 5.1. Operating Phases

**Figure 11:**
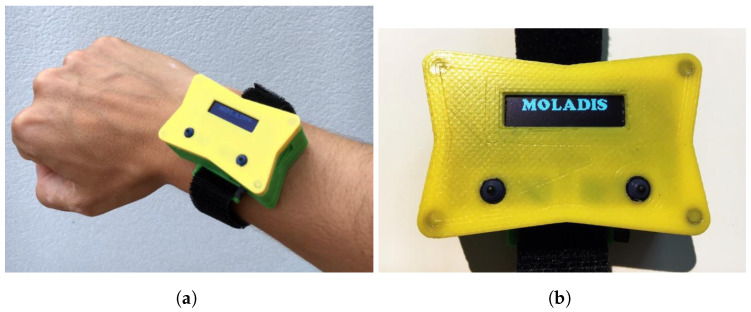
Device placement (**a**) and standby screen (**b**).

**Figure 12:**
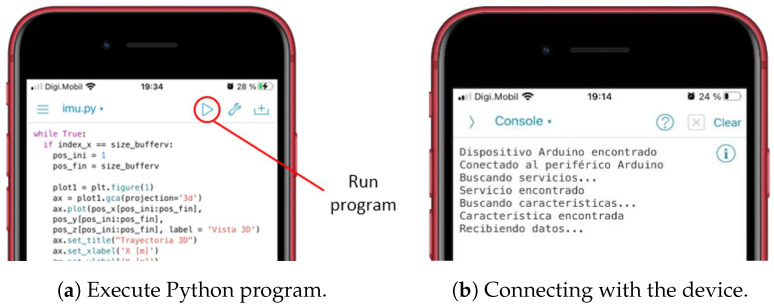
Execution of program (**a**) and connection to the device (**b**).

**Figure 13:**
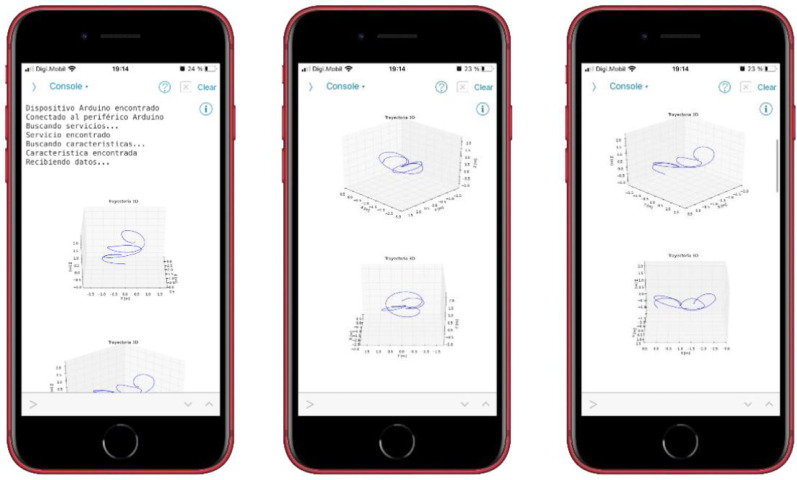
Receiving data from the device.

The process, from start to finish, is:

1. Placement of the device: The athlete must firmly hold the device to the wrist using the tape that is incorporated. If it is not properly secured, it can cause vibrations, which generate incorrect readings in the IMU; see Figure 11a.
2. Power on: To power on the device, it is necessary to move the slide switch to the right. When it is turned on, the text “MOLADIS” appears on the screen (Figure 11b).
3. Start monitoring: To start capturing the data from IMU, the left button should be pressed; see Figure 11b.
4. The text “Stabilization” appears on the screen together with a countdown. This countdown corresponds to the stabilization time of the calculations made by the fusion algorithm and it also helps the athlete to prepare.
5. Once the countdown has finished, the device will make a small vibration to indicate that the data acquisition by the has IMU begun. In addition, the text “Capturing movement” appears on the screen.
6. At this point, the athlete should begin the movement he/she wants to monitor. The data collection is carried out during 6 s, where 3000 measurements are stored. When this process is finished, the device performs another vibration with a different pattern than the previous one to indicate that the data capture has finished.
7. Once the capture has finished, the microcontroller processes the collected information. The text “Computing speed and positions” appears on the screen.
8. After the processing, the data are sent to the smart phone via Bluetooth. This sending phase is indicated on the screen with the text “Waiting for Bluetooth connection”.
9. When the previous message appears, it is time to run the application for the data reception and display on the smart phone. While the information is being sent, the message “Sending data” is shown.
10. On the smart phone, it is necessary to execute the program to receive the information. To do this, the execute button within the Pythonista program should be pressed; see Figure 12a.
11. The program runs in console mode. If the connection to the device is successful, it will first connect to the device, then search for a service and then a feature of the device; see Figure 12b. If it finds the feature correctly, the device starts transmitting the data, which are received by the smart phone.
12. When the information is received and interpreted by the program, the graphs appear, as shown in the screenshots of Figure 13.
  The graphs, which were described in Section 4.2.2, are: representation of the movement of the athlete’s hand in 3D and top and side view of the movement of the athlete’s hand in 2D. These latter representations offer a complementary vision to those of 3D. In addition, the speed of the athlete’s hand is also shown, representing the instantaneous speed of the athlete’s hand during the execution of the movement.

At this point, it is possible to take another capture by pressing the right button of the device. The countdown will appear again for the athlete to get ready, and the cycle will start again.

### 5.2. Results

**Figure 14:**
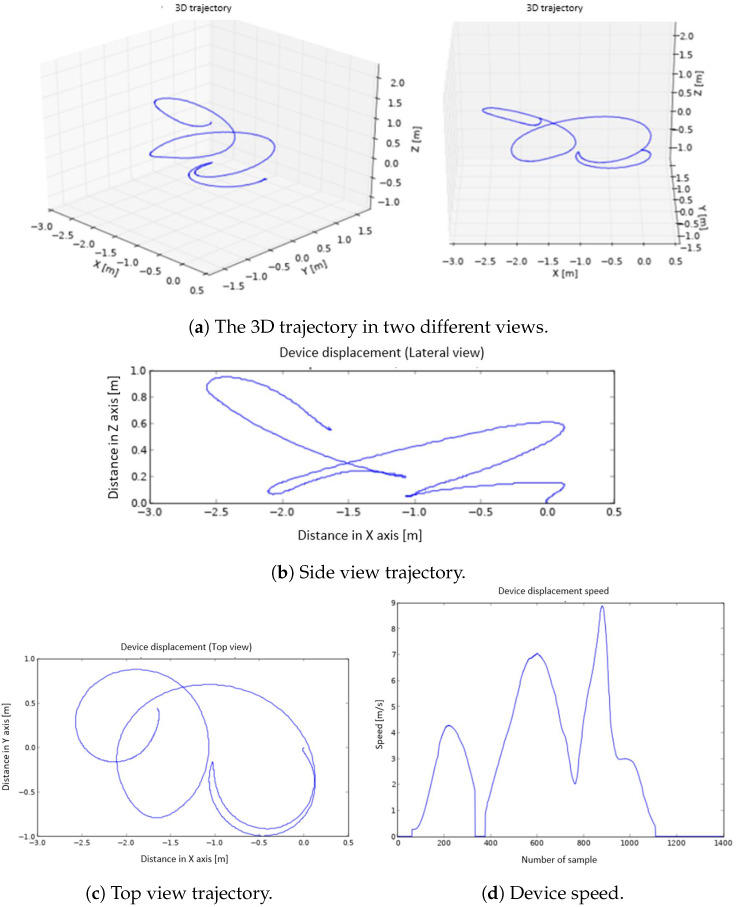
The 3D graphs (**a**), side view (**b**), top view (**c**), and device speed (**d**) representations.

As mentioned in previous sections, after the discus throwing, a series of graphs are generated on the smart phone. Among the several graphs plotted, the 3D trajectory graphs are the ones that allow the movement to be visualized in a more adequate way, and they are generated first. The graphs of the movement in top view, side view, and speed are shown below the 3D graphs; see Figure 14. In the speed graph, three well-defined peaks are observed. The first of them corresponds to the movement of half an arc of a circumference carried out in the opposite direction of the rest of the throw movement. The algorithm to correct the non-zero speed when the device is stopped (described at Point 8 in Section 4.1.3) adequately detects the direction change, since, for a small time window, the acceleration approaches 0, forcing the velocity to be 0 at this point. As a consequence, the trajectory computation is very precise and the change of direction is correctly described, which can be observed in the top view. The trajectory made, the rotation radius (displayed in the top view), and the elevation of the arm during the launch (side view) are very well defined; see Figure 14b,c. In the speed graph (Figure 14d), it can be seen how the highest value matches the exact moment of the discus throw.

These good results can be checked in other attempts carried out with the device and that have been used to illustrate the design, construction, and programming of the wearable embedded system. These tests were carried out by a small set of amateur athletes with experience with and knowledge of discus throwing. They performed multiple attempts, and three of these trials were used to illustrate the performance of the device. These additional throws can be seen in Figure 3 and Figure 10, where a similar behaviour to that explained in Figure 14 can be noticed.

It is difficult to offer a comparison between the 3D trajectories recorded with this low-cost system and the data found in papers from the sports and biomechanics literature. The reason is that these papers use information from Olympic and elite athletes and also record data at specific time instants (for example, angles, speeds, and accelerations at the release of the discus), not the path, speed, and acceleration of the wrist’s athlete during the phases of the throw. However, and when possible, we checked if the trajectories recorded with the amateur athletes were in the range of some of the data found in specialized papers [3,21], and the results agree, obviously, taking into consideration the differences between elite and amateur athletes.

## 6. Conclusions

In this work, a low-cost embedded system was developed to capture the physical variables related to the movement of an athlete’s hand and, subsequently, represent the trajectory and speed of the athlete’s hand on a smart phone. In particular, the representation of the trajectory described by the athlete’s wrist during the phases of the discus throwing, both in 2D and 3D, and the hand’s speed are shown in the mobile phone app just after the throwing. The tests carried out for its verification provided satisfactory and acceptable results, showing that the device can be used to monitor and track the training of the athletes. Moreover, an important feature to consider in this solution, and that is not present in typical high-speed video recording and data processing systems used in the sports literature [3,19,20,21,22,23,24], is the recording and plotting of 3D trajectories in real-time of some kinematics parameters of a body part (in this work, the athlete’s wrist).

The development of the microcontroller software that manages the IMU meant overcoming a series of challenges. Among them can be noted:

- The sensor calibration and data fusion algorithm implementation.
- The measurements’ filtering.
- The correction of the drift problem caused by the gyroscopes.
- The error correction in the calculations caused by the integration of accelerations and speeds.
- The use of quaternions instead of Euler angles to avoid the gimbal lock.
- The high speed of execution of the reading of the sensors and the transmission of the data arrays through BLE.

It must be noticed that the use of this type of device to represent the movement of a mobile entails a series of limitations that should be considered when validating the results obtained. The main limitation is that long-term records cannot be made without some reset mechanism (dead reckoning) [31], or having an external reference (GPS signal, artificial vision) to correct the measurement errors. Without these mechanisms, the measurement drift caused by the gyroscopes and/or the error accumulated during the integration, first of the acceleration and then of the speed, would make the acquisition of data during a long time useless. For this work, since it involves acquisitions of short movements, it is not necessary to use auxiliary mechanisms or external references.

After multiple tests carried out with the device, it is clear that the correct calibration of the sensors is of the utmost importance. The magnetometers are the sensors that need a more precise calibration, and their measurements were drastically different before and after placing the IMU inside the casing. Accelerometers and gyroscopes also needed a new calibration once the IMU was placed inside the casing, but their errors were of a smaller magnitude than the magnetometer errors.

For future works, additional measurement systems will be added to the sports equipment used by the athletes, so as to monitor the use given to the equipment and also to compare with a gold standard, so as to check the reliability of the device constructed. In addition, location sensor networks, based on previous works by the authors [32,33], will be installed in the wearable embedded system and the discus throw area to obtain a more precise positioning of the athlete’s hand.

## Figures and Tables

**Table 1:** Sensors’ full scale.

Sensor	Full Scale	Resolution
Accelerometer	±8 g	0.244 mg/LSB
Gyroscope	±2000 dps	70 mdps/LSB
Magnetometer	±8 gauss	0.29 mgauss/LSB

**Table 2:** Sensors output data rate (ODR).

Sensor	ODR
Accelerometer	476 Hz
Gyroscope	476 Hz
Magnetometer	40 Hz

## Appendix A. Calibration

The negative effect of the measurement errors is minimized with the correct calibration. The calibration and adaptation of the sensors measurements is performed in the microcontroller after reading the raw data [25]:

- Gyroscope calibration: The simplest way to calibrate the gyroscope is to compute the offset in each of the axes. The measurement in each axis must be zero degrees per second if the IMU is in the resting position. The offset computed will be used to correct the measurements once the IMU is moving. This process is valid in most applications [29].
- Accelerometer calibration: Knowing the direction and intensity of the gravity acceleration, the accelerometer can be calibrated correctly in each of the axes [29]. Each axis will be oriented for a positive maximum value, a negative minimum value, and a zero value of the gravity acceleration, providing three measurements per axis, which will be used to calibrate the accelerometers.
- Magnetometer calibration: For the correct calibration of the magnetometer, it is important that the IMU be inside the casing, that is it must be done once the device is constructed and mounted. This will allow correcting the distortions caused by hard and soft iron [29,34]. During the calibration process, it is necessary to rotate the device in all directions while the magnetic field lines that pass through the device are measured. It is necessary to know the angle of the magnetic field of the place in which the calibration is carried out. Using this angle and the measurements of the magnetic field lines, the offset and slope needed to correct the distortions in each axis are computed. For the calibration of the magnetometer, the first step is to set the full scale to be ±800μT and the sensor frequency to 414 Hz. The magnetic field intensity, which depends on the location of the sensors, must also be set. This value is obtained by the National Centre for Environmental Information, National Oceanic and Atmospheric Administration (NOAA) [35]. Once the parameters are configured, the device is rotated until the values of the magnetic field remain stable. At this moment, the parameters to calibrate the sensors can be obtained.

## References

[1] Lara O.D., Labrador M.A. (2013). A Survey on Human Activity Recognition using Wearable Sensors. IEEE Commun. Surv. Tutor..

[2] De-La-Hoz-Franco E., Ariza-Colpas P., Quero J.M., Espinilla M. (2018). Sensor-Based Datasets for Human Activity Recognition—A Systematic Review of Literature. IEEE Access.

[3] Bartlett R.M. (1992). The biomechanics of the discus throw: A review. J. Sports Sci..

[4] Kos A., Milutinović V., Umek A. (2019). Challenges in wireless communication for connected sensors and wearable devices used in sport biofeedback applications. Future Gener. Comput. Syst..

[5] Xie X. (2022). Real-Time Monitoring Of Big Data Sports Teaching Data Based On Complex Embedded System. Microprocess. Microsyst..

[6] Pierleoni P., Raggiunto S., Marzorati S., Palma L., Cucchiarelli A., Belli A. (2022). Activity Monitoring Through Wireless Sensor Networks Embedded Into Smart Sport Equipments: The Nordic Walking Training Utility. IEEE Sens. J..

[7] Ghasemzadeh H., Loseu V., Guenterberg E., Jafari R. Sport Training Using Body Sensor Networks: A Statistical Approach to Measure Wrist Rotation for Golf Swing. Proceedings of the Fourth International Conference on Body Area Networks BodyNets ’09, ICST (Institute for Computer Sciences, Social-Informatics and Telecommunications Engineering).

[8] Bornand C., Güsewell A., Staderini E., Patra J. Sport and Technology: The Case of Archery. Proceedings of the 2013 Humaine Association Conference on Affective Computing and Intelligent Interaction.

[9] Xu W., Liu F. (2021). Design of embedded system of volleyball training assistant decision support based on association rules. Microprocess. Microsyst..

[10] Hölzemann A., Van Laerhoven K. (2018). Using Wrist-Worn Activity Recognition for Basketball Game Analysis. Proceedings of the 5th International Workshop on Sensor-Based Activity Recognition and Interaction iWOAR ’18.

[11] Zhou J. (2021). Virtual reality sports auxiliary training system based on embedded system and computer technology. Microprocess. Microsyst..

[12] Huang C., Jiang L. (2021). Data monitoring and sports injury prediction model based on embedded system and machine learning algorithm. Microprocess. Microsyst..

[13] Peng Y. (2021). Research on teaching based on tennis-assisted robot image recognition. Microprocess. Microsyst..

[14] Kerdjidj O., Boutellaa E., Amira A., Ghanem K., Chouireb F. (2022). A hardware framework for fall detection using inertial sensors and compressed sensing. Microprocess. Microsyst..

[15] Ferrand S., Alouges F., Aussal M. (2020). An electronic travel aid device to help blind people playing sport. IEEE Instrum. Meas. Mag..

[16] Nemstev O. (2011). Comparison of kinematic characteristics between standing and rotational discus throwss. Porto Int. Soc. Biomech. Sports.

[17] Panoutsakopoulos V., Kollias I. (2012). Temporal analysis of elite men’s discus throwing technique. J. Hum. Sport Exerc..

[18] Chen C.F., Wu H.J., Yang Z.S., Chen H., Peng H.T. (2021). Motion Analysis for Jumping Discus Throwing Correction. Int. J. Environ. Res. Public Health.

[19] Leigh S., Liu H., Hubbard M., Yu B. (2010). Individualized optimal release angles in discus throwing. J. Biomech..

[20] Gregor R.J., Whiting W.C., McCoy R.W. (1985). Kinematic Analysis of Olympic Discus Throwers. Int. J. Sport Biomech..

[21] Hay J.G., Yu B. (1995). Critical characteristics of technique in throwing the discus. J. Sports Sci..

[22] Leigh S., Gross M.T., Li L., Yu B. (2008). The relationship between discus throwing performance and combinations of selected technical parameters. Sports Biomech..

[23] Wang L., Hao L., Zhang B. (2022). Kinematic Diagnosis of Throwing Motion of the Chinese Elite Female Discus Athletes Who Are Preparing for the Tokyo Olympic Games. J. Environ. Public Health.

[24] Yu B., Broker J., Silvester L.J. (2002). Athletics. Sports Biomech..

[25] Leibson S. (2019). IMUs for Precise Location. Contributed By Digi-Key’s North American Editors. https://www.digikey.es/en/articles/imus-for-precise-location-part-2-how-to-use-imu-software-for-greater-precision.

[26] Chérigo C., Rodríguez H. (2017). Evaluación De Algoritmos De Fusión De Datos Para Estimación De La Orientación De Vehículos Aéreos No Tripulados. I+D Tecnol..

[27] Madgwick S. (2010). An efficient orientation filter for inertial and inertial / magnetic sensor arrays. Rep. x-io Univ. Bristol (UK).

[28] Kong X. (2004). INS algorithm using quaternion model for low cost IMU. Robot. Auton. Syst..

[29] Hrisko J. (2021). Gyroscope and Accelerometer Calibration with Raspberry Pi.

[30] Garcés D. (2018). Estudio e Implementación de Algoritmos Para la Estimación de la Posición Mediante Sistemas Inerciales con Arduino. Ph.D. Thesis.

[31] Yin H., Guo H., Deng X., Yu M., Xiong J. Pedestrian Dead Reckoning Indoor Positioning with Step Detection Based on Foot-mounted IMU. Proceedings of the 2014 International Technical Meeting of The Institute of Navigation.

[32] Moreno-Salinas D., Pascoal A., Aranda J. (2013). Optimal Sensor Placement for Multiple Target Positioning with Range-Only Measurements in Two-Dimensional Scenarios. Sensors.

[33] Moreno-Salinas D., Pascoal A., Aranda J. (2013). Sensor Networks for Optimal Target Localization with Bearings-Only Measurements in Constrained Three-Dimensional Scenarios. Sensors.

[34] FAQ: Hard & Soft Iron Correction for Magnetometer Measurements. https://ez.analog.com/mems/w/documents/4493/faq-hard-soft-iron-correction-for-magnetometer-measurements.

[35] National Centre for Environmental Information, National Oceanic and Atmospheric Administration (NOAA). https://www.ngdc.noaa.gov/geomag/.

